# Analytical Solutions to the Unsteady Poiseuille Flow of a Second Grade Fluid with Slip Boundary Conditions

**DOI:** 10.3390/polym16020179

**Published:** 2024-01-07

**Authors:** Evgenii S. Baranovskii

**Affiliations:** Department of Applied Mathematics, Informatics and Mechanics, Voronezh State University, 394018 Voronezh, Russia; esbaranovskii@gmail.com

**Keywords:** Poiseuille flow, Rivlin–Ericksen fluids, second grade fluids, slip boundary conditions, analytical solutions, existence and uniqueness theorem, Sturm–Liouville problem, Abel’s criteria

## Abstract

This paper deals with an initial-boundary value problem modeling the unidirectional pressure-driven flow of a second grade fluid in a plane channel with impermeable solid walls. On the channel walls, Navier-type slip boundary conditions are stated. Our aim is to investigate the well-posedness of this problem and obtain its analytical solution under weak regularity requirements on a function describing the velocity distribution at initial time. In order to overcome difficulties related to finding classical solutions, we propose the concept of a generalized solution that is defined as the limit of a uniformly convergent sequence of classical solutions with vanishing perturbations in the initial data. We prove the unique solvability of the problem under consideration in the class of generalized solutions. The main ingredients of our proof are a generalized Abel criterion for uniform convergence of function series and the use of an orthonormal basis consisting of eigenfunctions of the related Sturm–Liouville problem. As a result, explicit expressions for the flow velocity and the pressure in the channel are established. The constructed analytical solutions favor a better understanding of the qualitative features of time-dependent flows of polymer fluids and can be applied to the verification of relevant numerical, asymptotic, and approximate analytical methods.

## 1. Introduction

It is well known that simulations of flows of polymer solutions and melts produce many challenging mathematical problems. The widespread use of polymeric materials in numerous industrial and engineering fields requires an elaborate mathematical theory of polymer media. In the past few decades, different complex models of polymer dynamics have been proposed (see [1,2,3,4] and the references therein), and it cannot be said that a particular model is dominant over others.

Many polymer fluids belong to the class of *fluids of complexity N*, which obey the following constitutive relation (see [5,6]):

$$T=-pI+F(A_{1},\ldots ,A_{N}),$$

where

-
T is the Cauchy stress tensor;
-
I is the identity tensor;
-
F is a frame indifferent response function;
-
$A_{1},\ldots ,A_{N}$ are the first *N* Rivlin–Ericksen tensors:
$$\begin{array}{cc} A_{1} & \overset{\mathrm{def}}{=}\nabla v+{\left(\nabla v\right)}^{⊤},\\ A_{j} & \overset{\mathrm{def}}{=}\frac{d}{dt}A_{j-1}+A_{j-1}\nabla v+{\left(\nabla v\right)}^{⊤}A_{j-1},\;j=2,\ldots ,N; \end{array}$$
-
v is the flow velocity;
-
∇v denotes the velocity gradient;
-
${\left(\nabla v\right)}^{⊤}$ denotes the transpose of the velocity gradient;
- the differential operator d/dt is the material time derivative defined by
$$\frac{d}{dt}S\overset{\mathrm{def}}{=}\frac{\partial }{\partial t}S+\left(v\cdot \nabla \right)S;$$
- *p* is the pressure.

Recall that if F is a polynomial of degree *N*, then the fluid is called a *fluid of grade N*.

The classical incompressible Newtonian fluid satisfies the constitutive relation

$$T=-pI+\mu A_{1},\;\mu =\mathrm{const}>0,$$

and is a fluid of grade 1, while fluids with shear-dependent viscosity modeling by

$$T=-pI+\mu \left(A_{1}\right)A_{1},\;\mu \left(A_{1}\right)>0,$$

belong to the class of fluids of complexity 1.

In this paper, we deal with the second grade fluids:

(1) $$T=-pI+\mu A_{1}+\alpha_{1}A_{2}+\alpha_{2}A_{1}^{2},$$

where

-
μ is the viscosity coefficient, μ>0 ;
-
$\alpha_{1}$ and $\alpha_{2}$ are the normal stress moduli.

Relation (1) is well suited to describe the dynamics of some nonlinear viscoelastic fluids, for example, diluted polymer suspensions.

Previous studies have shown that a viscoelastic fluid modeled by (1) is compatible with the thermodynamic laws and stability principles if the following restrictions hold (see [7,8]):

(2) $$\alpha_{1}\geq 0,\;\alpha_{1}+\alpha_{2}=0.$$

For more details on the physical background of fluids of differential type, we refer to the review paper [9].

In the sequel, it is assumed that both conditions from (2) are fulfilled. Using the notation

$$\alpha \overset{\mathrm{def}}{=}\alpha_{1}=-\alpha_{2},$$

one can rewrite relation (1) as follows:

(3) $$T=-pI+\mu A_{1}+\alpha A_{2}-\alpha A_{1}^{2}.$$

Clearly, a Newtonian fluid can be considered as the limit case of second grade fluids as $\alpha \to 0^{+}$ .

In this paper, we consider an initial-boundary value problem modeling the unsteady flow of a second grade fluid in a plane channel with impermeable solid walls. It is assumed that the flow is unidirectional and driven by a constant pressure gradient along the channel. Our main aim is to investigate the unique solvability of this problem and find its analytical solution under weak regularity requirements on a function describing the velocity distribution at initial time.

One of features of our work is that *Navier-type slip boundary conditions* [10] are used on the channel walls instead of the standard no-slip boundary condition v=0 . The importance of interfacial (slip) constitutive laws and their influence on various flow characteristics (especially for non-Newtonian fluid flows) are mentioned by many researchers (see, for example, [11,12,13,14,15,16] and the literature cited therein). In particular, as stated in [16], the study of wall slip is significant since it can be used to establish the true rheology of materials by correcting data related to slip effects and explaining mismatches in rheological data obtained from rheometers that have different geometries.

Another important feature of the present work is that analytical solutions are constructed not just for the motion equations with boundary conditions but for an *initial-boundary value problem* for these equations with initial data from a very wide class of functions.

The plan of the paper is as follows. In the next section, we give the preliminaries that are needed for mathematical handling of the problem. In Section 3, we provide a description of the mathematical model under consideration and rigorously formulate the initial-boundary value problem for the Poiseuille flow with slip boundary conditions. Section 4 is devoted to deriving an explicit expression for the pressure under the assumption that the velocity field is known. With this expression in hand, we can focus on finding the velocity field, which is performed in Section 5. We consider both classical and generalized formulations of an initial-boundary value boundary problem related to a third-order partial differential equation describing the flow velocity. It is shown that the classical solution is unique and satisfies an energy equality (Theorem 1). But, in general, the classical formulation is inconvenient because, in this framework, our problem is not solvable for a wide class of natural initial data. In order to overcome this difficulty, we introduce the concept of a generalized solution that is defined as the limit of a uniformly convergent sequence of classical solutions with vanishing perturbations in initial data. We prove the unique solvability of the problem under consideration in the class generalized solutions (Theorem 2). The main ingredients of our proof are a generalized Abel criterion for uniform convergence of function series (Proposition 3) and the use of an orthonormal basis consisting of eigenfunctions of the related Sturm–Liouville problem. As a result, we arrive at explicit formulas for the flow velocity and pressure in the channel, assuming that the initial velocity belongs to a suitable Sobolev space.

The present paper is a continuation of the works [17,18,19], in which various slip problems were considered for the case of steady-state unidirectional motion. It should be mentioned that analytical solutions for both steady and time-dependent flows of second grade fluids were investigated by many authors. Some exact solutions related to flows of this class of viscoelastic fluids through straight channels or pipes under a constant pressure gradient were obtained by Ting [20]. The main object of his work was to find the role of the material constant $\alpha_{1}$ . Ting in particular showed the unboundedness of solutions for the case $\alpha_{1}<0$ . For partial differential equations (PDEs) describing time-dependent shearing flows of second grade fluids with $\alpha_{1}<0$ , a qualitative theory (instability, uniqueness, and nonexistence theorems) was developed by Coleman et al. [21]. Analytical solutions for the flow velocity corresponding to the second problem of Stokes were obtained in work [22] by applying the Laplace transform technique. Using the Caputo–Fabrizio time fractional derivative, Fetecau et al. [23] performed an analytical investigation of the magnetohydrodynamic (MHD) flow of second grade fluids over a moving infinite flat plate. In work [24], it was shown that the governing equations for velocity and non-trivial shear stress related to some isothermal MHD unidirectional flows of second grade fluids in a porous medium have identical forms. Shankar and Shivakumara [25] investigated the temporal stability of the plane Poiseuille and Couette flows of a Navier–Stokes–Voigt-type viscoelastic fluid. Recently, Fetecau and Vieru [26] obtained the general solutions for MHD flows of incompressible second grade fluids between infinite horizontal parallel plates which are embedded in a porous medium. Analysis of the second grade hybrid nanofluid flow over a stretching flat plate is given in [27]. A semi-analytical approach for the investigation of rivulet flows of non-Newtonian fluids was developed in [28,29]. Finally, we mention mathematical studies devoted to the existence and uniqueness of solutions to PDEs describing second grade fluid flows [30,31,32,33,34,35,36] as well as to optimal flow control and controllability problems [37,38,39,40].

The number of articles about second grade fluids and other types of viscoelastic media is constantly increasing, and this provides deeper understanding of relevant physical effects and support for technological advances, in particular, in the polymer industry. On the other hand, the existing research and challenging problems in this field motivate the development of new approaches for PDE analysis.

## 2. Mathematical Preliminaries

In this section, we collect the auxiliary statements that are needed to obtain the main results.

### 2.1. Function Spaces

$C^{0}\left[0,h\right]$ denotes the space of all continuous real-valued functions defined on [0,h] and endowed with the max-norm

$${∥v∥}_{C^{0}\left[0,h\right]}\overset{\mathrm{def}}{=}\max_{y\in [0,h]}\left|v\left(y\right)\right|.$$

As usual, $H^{1}\left[0,h\right]$ denotes the Sobolev space $W^{1,2}\left[0,h\right]$ . The scalar product and the norm in this space are defined as follows:

$$\begin{array}{cc} {\left(v,w\right)}_{H^{1}\left[0,h\right]} & \overset{\mathrm{def}}{=}\int_{0}^{h}v\left(y\right)w\left(y\right)\;dy+\int_{0}^{h}v^{\prime }\left(y\right)w^{\prime }\left(y\right)\;dy,\\ {∥v∥}_{H^{1}\left[0,h\right]} & \overset{\mathrm{def}}{=}{\left(v,v\right)}_{H^{1}\left[0,h\right]}^{\frac{1}{2}}, \end{array}$$

where the symbol^′^ denotes the derivative with respect to *y*.

Recall that $H^{1}\left[0,h\right]$ is compactly embedded in $C^{0}\left[0,h\right]$ . Therefore, if ${\left\{w_{i}\right\}}_{i=1}^{\infty }\subset H^{1}\left[0,h\right]$ , $w_{0}\in H^{1}\left[0,h\right]$ , and

$$w_{i}\to w_{0}\mathrm{in}\mathrm{the}\mathrm{space}H^{1}\left[0,h\right]\mathrm{as}i\to \infty ,$$

then

$$w_{i}⇉w_{0}\mathrm{on}\mathrm{the}\mathrm{interval}\left[0,h\right]\mathrm{as}i\to \infty .$$

For κ>0 , we introduce the space $H_{\kappa }^{1}\left[0,h\right]$ , consisting of functions that belong to $H^{1}\left[0,h\right]$ with the following scalar product:

$${\left(v,w\right)}_{H_{\kappa }^{1}\left[0,h\right]}\overset{\mathrm{def}}{=}\int_{0}^{h}v^{\prime }\left(y\right)w^{\prime }\left(y\right)\;dy+\kappa v\left(h\right)w\left(h\right)+\kappa v\left(0\right)w\left(0\right).$$

It is easy to show that this scalar product is well defined and the associated Euclidean norm is equivalent to the standard $H^{1}$ -norm.

### 2.2. Special Basis Constructed from Eigenfunctions of a Sturm–Liouville Problem

Applying the approach from [41], one can derive the following statement.

**Proposition** **1** (Special orthonormal basis)**.** *Let κ>0 . Suppose that ${\left\{\lambda_{i}\right\}}_{i=1}^{\infty }$ is the increasing sequence of eigenvalues of the following Sturm–Liouville problem:*

$$\left\{\begin{array}{c} -v^{\prime \prime }\left(y\right)=\lambda v\left(y\right),\;\;y\in \left(0,h\right),\\ -v^{\prime }\left(0\right)+\kappa v\left(0\right)=0,\\ \;\;v^{\prime }\left(h\right)+\kappa v\left(h\right)=0, \end{array}\right.$$

*and ${\left\{v_{i}\right\}}_{i=1}^{\infty }$ is the associated sequence of eigenfunctions such that $∥v_{i}{∥}_{H_{\kappa }^{1}\left[0,h\right]}=1,$ for all i∈N . Then ${\left\{v_{i}\right\}}_{i=1}^{\infty }$ is an orthonormal basis of the space $H_{\kappa }^{1}\left[0,h\right]$ .*

### 2.3. Abel’s Criterion for Series of Functions of Various Variables

Let us recall one well-known statement arising in the theory of numerical series.

**Proposition** **2** (Abel’s lemma)**.** *Let $\alpha_{1},\ldots ,\alpha_{m},\beta_{1},\ldots ,\beta_{m}$ be real numbers and*

$$B_{s}\overset{\mathrm{def}}{=}\sum_{i=1}^{s}\beta_{i},\;\;s\in \left\{1,\ldots ,m\right\}.$$

*Suppose the sequence ${\left\{\alpha_{i}\right\}}_{i=1}^{m}$ is monotone and L is a number such that*

$$\max_{s\in \{1,\ldots ,m\}}\left|B_{s}\right|\leq L,$$

*then*

$$\left|\sum_{i=1}^{m}\alpha_{i}\beta_{i}\right|\leq L(|\alpha_{1}\left|+2\right|\alpha_{m}\left|\right).$$

Below, we present a generalization of Abel’s uniform convergence test for the case where functions depend on various variables.

**Proposition** **3** (Generalized Abel’s criterion)**.** *Let X and Y be subsets of the set of real numbers.*

*Suppose the functions $a_{n}:\;X\to R$ and $b_{n}:\;Y\to R$ , with n∈N , satisfy the following three conditions:*

(C.1) *the sequence ${\left\{a_{n}\left(x\right)\right\}}_{n=1}^{\infty }$ is monotone for any x∈X ;*
(C.2) *there exists a number K such that $|a_{n}\left(x\right)|\leq K$ for any x∈X and n∈N;
*
(C.3) *the function series $\sum_{n=1}^{\infty }b_{n}\left(y\right)$ is uniformly convergent on the set Y.
*

*Then the function series $\sum_{n=1}^{\infty }a_{n}\left(x\right)b_{n}\left(y\right)$ is uniformly convergent on the Cartesian product X×Y .*

**Proof.**  Let

$$\begin{array}{cc} {\tilde{\alpha }}_{n,i}\left(x\right)\overset{\mathrm{def}}{=}a_{n+i}\left(x\right), & \;{\tilde{\beta }}_{n,i}\left(y\right)\overset{\mathrm{def}}{=}b_{n+i}\left(y\right),\\ B_{n,i}\left(y\right)\overset{\mathrm{def}}{=}\sum_{j=1}^{i}{\tilde{\beta }}_{n,j}\left(y\right), & \;S_{n,m}\left(x,y\right)\overset{\mathrm{def}}{=}\sum_{j=n+1}^{n+m}a_{j}\left(x\right)b_{j}\left(y\right), \end{array}$$

for any n, m, i∈N , x∈X , and y∈Y . Note that

(4) $$\begin{array}{cc} S_{n,m}\left(x,y\right) & =\sum_{i=1}^{m}a_{n+i}\left(x\right)b_{n+i}\left(y\right)\\ \; & =\sum_{i=1}^{m}{\tilde{\alpha }}_{n,i}\left(x\right){\tilde{\beta }}_{n,i}\left(y\right). \end{array}$$

In view of condition (C.3), for an arbitrary positive number ε , there exists an integer $N_{\varepsilon }$ such that

$$\left|\sum_{j=1}^{m}b_{n+j}\left(y\right)\right|<\frac{\varepsilon }{3K},$$

for any $n\in \{N_{\varepsilon },\;N_{\varepsilon }+1,\;N_{\varepsilon }+2,\ldots \}$ , m∈N , and y∈Y .

Therefore, we have

$$\begin{array}{cc} |B_{n,i}\left(y\right)| & =\left|\sum_{j=1}^{i}{\tilde{\beta }}_{n,j}\left(y\right)\right|\\ \; & =\left|\sum_{j=1}^{i}b_{n+j}\left(y\right)\right|\leq \frac{\varepsilon }{3K}, \end{array}$$

for any $n\in \{N_{\varepsilon },\;N_{\varepsilon }+1,\;N_{\varepsilon }+2,\ldots \}$ , i∈N , and y∈Y .

Moreover, from condition (C.1) it follows that, for any n∈N and x∈X , the sequence ${\left\{{\tilde{\alpha }}_{n,i}\left(x\right)\right\}}_{i=1}^{\infty }$ is monotone.

Using Abel’s lemma, condition (C.2), and (4), we derive

$$\begin{array}{cc} |S_{n,m}\left(x,y\right)| & =\left|\sum_{i=1}^{m}{\tilde{\alpha }}_{n,i}\left(x\right){\tilde{\beta }}_{n,i}\left(y\right)\right|\\ \; & \leq \frac{\varepsilon }{3K}\left(|\alpha_{n,1}\left(x\right)|+2|\alpha_{n,m}\left(x\right)|\right)\\ \; & =\frac{\varepsilon }{3K}\left(|a_{n+1}\left(x\right)|+2|a_{n+m}\left(x\right)|\right)\leq \varepsilon , \end{array}$$

for any $n\in \{N_{\varepsilon },\;N_{\varepsilon }+1,\;N_{\varepsilon }+2,\ldots \}$ , m∈N , and (x,y)∈X×Y . This yields that the function series $\sum_{n=1}^{\infty }a_{n}\left(x\right)b_{n}\left(y\right)$ is uniformly convergent on the set X×Y . Thus, Proposition 3 is proved. □

## 3. Description of the Mathematical Model

### 3.1. Flow Configuration

We will consider the unidirectional motion of a second grade fluid between the horizontal parallel plates y=0 and y=h , assuming that the flow is driven by a constant pressure gradient

(5) $$\frac{\partial p}{\partial x}=-\xi ,\;\xi =\mathrm{const},\;\xi >0.$$

In other words, we deal with the *plane Poiseuille flow*.

**Figure 1:**
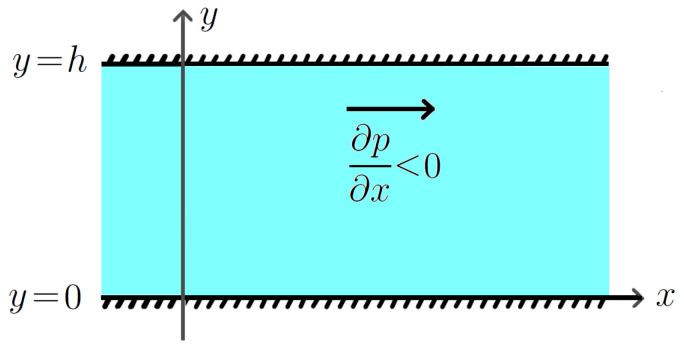
Sketch of the plane Poiseuille flow.

Figure 1 shows the chosen coordinate system and the flow geometry.

### 3.2. Governing Equations

As is well known, the unsteady flow of a fluid with constant density is governed by the following system of equations:

(6) $$\begin{array}{cc} \; & \mathrm{The}\mathrm{momentum}\mathrm{equation}:\;\rho \left(\frac{\partial v}{\partial t}+\left(v\cdot \nabla \right)v\right)=\mathrm{div}T+\rho g, \end{array}$$

(7) $$\begin{array}{cc} \; & \mathrm{equation}\mathrm{continuity}\mathrm{equation}:\;\;\;\nabla \cdot v=0, \end{array}$$

where there are the following variables:

-
ρ is the fluid density, ρ>0 ;
-
$v={\left(v_{1},v_{2},v_{3}\right)}^{⊤}$ is the velocity vector;
-
T is the Cauchy stress tensor;
-
$g={\left(g_{1},g_{2},g_{3}\right)}^{⊤}$ is the external force per unit mass;
- the operators div and ∇ are the divergence and the gradient, respectively (with respect to the space variables *x*, *y*, *z*).

Assume that

(8) $$g={\left(0,-g,0\right)}^{⊤},$$

where *g* is the value of acceleration due to gravity.

Within the framework of the Poiseuille flow, for the velocity components $v_{1}$ , $v_{2}$ , $v_{3}$ , we have

$$v_{1}=u,\;v_{2}=0,\;v_{3}=0,$$

where u=u(y,t) is an unknown function. Then the following equalities hold:

(9) ∇·v=0,(v·∇)v=0.

In view of the second relation from (9), Equation (6) reduces to

(10) $$\begin{array}{c} \rho \frac{\partial v}{\partial t}=\mathrm{div}T+\rho g. \end{array}$$

For simplicity, we assume the density ρ is equal to 1. Since the fluid obeys the constitutive relation (3), we can rewrite (10) in the form

(11) $$\frac{\partial v}{\partial t}=\mathrm{div}\left(\mu A_{1}+\alpha A_{2}-\alpha A_{1}^{2}\right)-\nabla p+g.$$

### 3.3. Statement of Initial-Boundary Value Problem for the Poiseuille Flow

Let us introduce the notation:

$$\Pi_{h}\overset{\mathrm{def}}{=}\left\{\left(x,y,z\right)\in R^{3}:\;0<y<h\right\},\;\Gamma_{a}\overset{\mathrm{def}}{=}\left\{\left(x,y,z\right)\in R^{3}:\;y=a\right\}.$$

We use (11) for handling the unsteady Poiseuille flow of a second grade fluid in the channel $\Pi_{h}$ at a given time interval [0,T] . Of course, in order to obtain physically relevant solutions, Equation (11) must be supplemented with suitable boundary and initial conditions for the velocity field. Taking into account slip effects, we arrive at the following initial-boundary value problem for the Poiseuille flow (IBVP-PF).

*For a given function $u_{0}:\;\left[0,h\right]\to R$ (initial data), find functions u: [0,h]×[0,T]→R and $p:\;{\bar{\Pi }}_{h}\times \left[0,T\right]\to R$ such that $v={\left(u,0,0\right)}^{⊤}$ and p satisfy* (5) *and* (11) *in $\Pi_{h}\times \left(0,T\right)$ and the following three conditions hold:*

(i) *the impermeability boundary condition*
(12) $$v\cdot n=0\;on\;\left(\Gamma_{0}\cup \Gamma_{h}\right)\times \left(0,T\right);$$
(ii) *the slip boundary condition*
(13) $$\mu {\left(A_{1}n\right)}_{\tan}=-kv_{\tan}\;on\;\left(\Gamma_{0}\cup \Gamma_{h}\right)\times \left(0,T\right),$$
  *where n is the unit outward normal vector to the channel walls and k is the slip coefficient, k>0;
*
(iii) *the initial condition*
(14) $${v|}_{t=0}={\left(u_{0},0,0\right)}^{⊤}\;in\;\Pi_{h}.$$

Section 4 and Section 5 are devoted to finding an analytical solution to the IBVP-PF.

Note that the limit case of boundary condition (13) when k=0 corresponds to the perfect slip condition [42,43,44,45], which means that the fluid actually does not interact with the walls of the channel. On the contrary, if k→+∞ , then relations (12) and (13) tend to the classical no-slip boundary condition v=0 . Thus, Navier’s slip boundary condition can be considered a homotopy transformation connecting the no-slip condition on the one hand with the perfect slip condition on the other hand. For modeling slip effects on surfaces that have portions with different physical properties, it is convenient to use a variable slip coefficient. In this regard, see, for example, the work [46], dealing with a position-dependent Navier-type slip boundary condition, which is formulated in terms of the operator **curl**.

## 4. Explicit Expression for the Pressure in Terms of the Velocity Gradient

First, we calculate the Rivlin–Ericksen tensors $A_{1}$ and $A_{2}$ :

$$A_{1}=\left[\begin{array}{ccc} 0\; & \frac{\partial u}{\partial y}\; & 0\\ \frac{\partial u}{\partial y}\; & 0\; & 0\\ 0\; & 0\; & 0 \end{array}\right],\;A_{2}=\left[\begin{array}{ccc} 0\; & \frac{\partial^{2}u}{\partial y\partial t}\; & 0\\ \frac{\partial^{2}u}{\partial y\partial t}\; & 2{\left(\frac{\partial u}{\partial y}\right)}^{2}\; & 0\\ 0\; & 0\; & 0 \end{array}\right].$$

Next, using these equalities, we rewrite (11) in the form

$$\frac{\partial v}{\partial t}=\mathrm{div}\left[\begin{array}{ccc} -\alpha {\left(\frac{\partial u}{\partial y}\right)}^{2}\; & \mu \frac{\partial u}{\partial y}+\alpha \frac{\partial^{2}u}{\partial y\partial t}\; & 0\\ \mu \frac{\partial u}{\partial y}+\alpha \frac{\partial^{2}u}{\partial y\partial t}\; & \alpha {\left(\frac{\partial u}{\partial y}\right)}^{2}\; & 0\\ 0\; & 0\; & 0 \end{array}\right]-\nabla p+g.$$

Taking into account (8), it is easy to see that the last equation is equivalent to the following system:

(15) $$\begin{array}{cc} \frac{\partial u}{\partial t}-\mu \frac{\partial^{2}u}{\partial y^{2}}-\alpha \frac{\partial^{3}u}{\partial y^{2}\partial t}+\frac{\partial p}{\partial x} & =\;0, \end{array}$$

(16) $$\begin{array}{cc} -\alpha \frac{\partial }{\partial y}\left[{\left(\frac{\partial u}{\partial y}\right)}^{2}\right]+\frac{\partial p}{\partial y} & =-g, \end{array}$$

(17) $$\begin{array}{cc} \frac{\partial p}{\partial z} & =\;0. \end{array}$$

Note that (15)–(17) can be considered as a starting point for solving the IBVP-PF.

Now, we are ready to find the pressure *p* under the assumption that the velocity field is known. In view of (17), the pressure is independent of *z*, that is, p=p(x,y,t) . Moreover, taking into account (5), we conclude that the pressure should be sought in the form

(18) p(x,y,t)=−ξx+ϕ(y,t)

with an unknown function ϕ=ϕ(y,t) .

From (16) and (18), it follows that

(19) $$\phi \left(y,t\right)=\alpha {\left(\frac{\partial u}{\partial y}\right)}^{2}-gy+C\left(t\right),$$

where C=C(t) is an arbitrary function.

Substituting (19) into (18), we obtain the resulting formula for the pressure in the channel:

(20) $$p\left(x,y,t\right)=-\xi x+\alpha {\left(\frac{\partial u}{\partial y}\right)}^{2}-gy+C\left(t\right).$$

**Remark** **1.** *In the next section, we obtain an explicit formula for the function u, and then one can return to* (20) *for the calculation of the pressure p.*

## 5. Initial-Boundary Value Problem Related to the Flow Velocity

In order to find the *x*-component of the velocity field, we must solve the initial-boundary value problem

(21) $$\left\{\begin{array}{cc} \frac{\partial u}{\partial t}-\mu \frac{\partial^{2}u}{\partial y^{2}}-\alpha \frac{\partial^{3}u}{\partial t\partial y^{2}}=\xi ,\; & \;y\in (0,h),\;t\in (0,T),\\ \mu \frac{\partial u}{\partial y}=ku,\; & \;y=0,\;\;t\in (0,T),\\ \mu \frac{\partial u}{\partial y}=-ku,\; & \;y=h,\;\;t\in (0,T),\\ u=u_{0},\; & \;y\in (0,h),\;\;t=0. \end{array}\right.$$

For the derivation of system (21), we used (12)–(15) and the equality ∂p/∂x=−ξ in $\Pi_{h}\times \left(0,T\right)$ .

### 5.1. Classical Solutions

**Definition** **1** (Classical solution)**.** *We shall say that a function u:[0,h]×[0,T]→R is a classical solution of initial-boundary value problem* (21) *if the following two conditions hold:*

(i) *the functions u, $\frac{\partial u}{\partial t}$ , $\frac{\partial u}{\partial y}$ , $\frac{\partial^{2}u}{\partial y^{2}},$ $\frac{\partial^{3}u}{\partial t\partial y^{2}}$ belong to the space $C^{0}\left(\left[0,h\right]\times \left[0,T\right]\right);$
*
(ii) *the function u satisfies system* (21) *in the usual sense.*

It is clear that a necessary condition for the solvability of problem (21) in the classical formulation is the fulfillment of the following equalities:

$$\mu u_{0}^{\prime }\left(0\right)=ku_{0}\left(0\right),\;\mu u_{0}^{\prime }\left(h\right)=-ku_{0}\left(h\right),$$

which are sometimes called the *compatibility conditions.* However, these conditions can be omitted when the generalized formulation is used (see Section 5.2).

**Theorem** **1** (Uniqueness and energy equality for classical solutions)**.** *Let u be a classical solution of problem* (21). *Then this solution is unique and satisfies the energy equality*

(22) $$\begin{array}{cc} \; & \int_{0}^{h}u^{2}\left(y,t\right)\;dy+\alpha \int_{0}^{h}{\left(\frac{\partial u}{\partial y}\left(y,t\right)\right)}^{2}dy+\frac{\alpha k}{\mu }\left(u^{2}\left(h,t\right)+u^{2}\left(0,t\right)\right)\\ \; & \;+2k\int_{0}^{t}\left(u^{2}\left(h,s\right)+u^{2}\left(0,s\right)\right)ds+2\mu \int_{0}^{t}\int_{0}^{h}{\left(\frac{\partial u}{\partial y}\left(y,s\right)\right)}^{2}dy\;ds\\ & =2\xi \int_{0}^{t}\int_{0}^{h}u\left(y,s\right)dy\;ds+\int_{0}^{h}u_{0}^{2}\left(y\right)\;dy+\alpha \int_{0}^{h}{\left(u_{0}^{\prime }\left(y\right)\right)}^{2}dy+\frac{\alpha k}{\mu }\left(u_{0}^{2}\left(h\right)+u_{0}^{2}\left(0\right)\right), \end{array}$$

*for any t∈[0,T] .*

**Proof.**  Let $u_{1}$ and $u_{2}$ be classical solutions of problem (21) and $w\overset{\mathrm{def}}{=}u_{1}-u_{2}$ . It is easy to see that

(23) $$\frac{\partial w}{\partial t}-\mu \frac{\partial^{2}w}{\partial y^{2}}-\alpha \frac{\partial^{3}w}{\partial t\partial y^{2}}=0,\;y\in \left(0,h\right),\;t\in \left(0,T\right),$$

(24) $$\mu \frac{\partial w}{\partial y}=kw,\;y=0,\;t\in \left(0,T\right),$$

(25) $$\mu \frac{\partial w}{\partial y}=-kw,\;y=h,\;t\in \left(0,T\right),$$

(26) w=0,y∈(0,h),t=0.

Let us multiply both sides of (23) by *w* and then integrate the obtained equality with respect to *y* from 0 to *h*:

(27) $$\int_{0}^{h}\frac{\partial w}{\partial t}\;w\;dy=\mu \int_{0}^{h}\frac{\partial^{2}w}{\partial y^{2}}\;w\;dy+\alpha \int_{0}^{h}\frac{\partial^{3}w}{\partial t\partial y^{2}}\;w\;dy.$$

Using the relation ${\left(f^{2}\right)}^{\prime }=2ff^{\prime }$ and integration by parts, we derive from (27) that

$$\begin{array}{c} \frac{1}{2}\int_{0}^{h}\frac{\partial }{\partial t}\;\left(w^{2}\right)\;dy=-\mu \int_{0}^{h}{\left(\frac{\partial w}{\partial y}\right)}^{2}\;dy+\mu \frac{\partial w}{\partial y}\left(h,t\right)\;w\left(h,t\right)-\mu \frac{\partial w}{\partial y}\left(0,t\right)\;w\left(0,t\right)\\ -\frac{\alpha }{2}\int_{0}^{h}\frac{\partial }{\partial t}\left({\left(\frac{\partial w}{\partial y}\;\right)}^{2}\;\right)\;dy+\alpha \frac{\partial^{2}w}{\partial t\partial y}\left(h,t\right)\;w\left(h,t\right)-\alpha \frac{\partial^{2}w}{\partial t\partial y}\left(0,t\right)\;w\left(0,t\right). \end{array}$$

Taking into account boundary conditions (24) and (25), we obtain

$$\begin{array}{c} \frac{1}{2}\int_{0}^{h}\frac{\partial }{\partial t}\;\left(w^{2}\right)\;dy=-\mu \int_{0}^{h}{\left(\frac{\partial w}{\partial y}\right)}^{2}\;dy-kw^{2}\left(h,t\right)-kw^{2}\left(0,t\right)\\ -\frac{\alpha }{2}\int_{0}^{h}\frac{\partial }{\partial t}\left({\left(\frac{\partial w}{\partial y}\;\right)}^{2}\;\right)\;dy-\frac{\alpha k}{2\mu }\frac{\partial w^{2}}{\partial t}\left(h,t\right)-\frac{\alpha k}{2\mu }\frac{\partial w^{2}}{\partial t}\left(0,t\right). \end{array}$$

Integrating this equality with respect to *t* from 0 to τ and using (26), we arrive at the following inequality:

$$\int_{0}^{h}w^{2}\left(y,\tau \right)\;dy\leq 0,\;\;\tau \in \left[0,T\right],$$

which implies that *w* is the zero function. Thus, we have $u_{1}=u_{2}$ .

In order to derive the energy equality that holds for the classical solution, we multiply both parts of the first equality from system (21) by the function *u*. Further, applying the integration by parts formula and then integrating the obtained equality with respect to time from 0 to *t*, we arrive at (22). Thus, Theorem 1 is proved. □

### 5.2. Generalized Solutions

The proposed concept of classical solutions is inconvenient because, in this framework, problem (21) is not solvable for a wide class of natural initial data. For example, if

(28) $$u_{0}\in C^{1}\left[0,h\right]\setminus C^{2}\left[0,h\right],$$

then problem (21) has no classical solutions. Indeed, assuming that *u* is a classical solution, we have

$$u_{0}=u\left(\cdot ,0\right)\in C^{2}\left[0,h\right],$$

which contradicts (28). Therefore, we introduce the concept of generalized solutions and then prove the corresponding existence and uniqueness theorem.

**Definition** **2** (Generalized solution)**.** *We shall say that a function u: [0,h]×[0,T]→R is a generalized solution to problem* (21) *if there exist sequences ${\left\{u^{\left(m\right)}\right\}}_{m=1}^{\infty }$ and ${\left\{u_{0}^{\left(m\right)}\right\}}_{m=1}^{\infty }$ such that the following three conditions hold:*

(i) *for any m∈N , the function $u^{\left(m\right)}$ is a classical solution to the problem*
$$\left\{\begin{array}{cc} \frac{\partial v}{\partial t}-\mu \frac{\partial^{2}v}{\partial y^{2}}-\alpha \frac{\partial^{3}v}{\partial t\partial y^{2}}=\xi ,\; & \;y\in (0,h),\;t\in (0,T),\\ \mu \frac{\partial v}{\partial y}=kv,\; & \;y=0,\;\;t\in (0,T),\\ \mu \frac{\partial v}{\partial y}=-kv\; & \;y=h,\;\;t\in (0,T),\\ v=u_{0}^{\left(m\right)}\; & \;y\in (0,h),\;\;t=0; \end{array}\right.$$
(ii) *the sequence ${\left\{u^{\left(m\right)}\right\}}_{m=1}^{\infty }$ converges to the function u uniformly on the set [0,h]×[0,T] as m→∞;
*
(iii) *the sequence ${\left\{u_{0}^{\left(m\right)}\right\}}_{m=1}^{\infty }$ converges to the function $u_{0}$ in the Sobolev space $H^{1}\left[0,h\right]$ as m→∞ .*

Clearly, if a function *u* is a classical solution to problem (21), then this function is a generalized solution to the one. The converse statement is not true. For example, a generalized solution can have smoothness that is not enough to be a classical solution.

The concept of generalized solutions has a clear physical meaning: a generalized solution is the uniform limit of some sequence of classical solutions of the model under consideration with small perturbations in the initial velocity data when these perturbations uniformly tend to the zero function.

The following theorem gives our main results.

**Theorem** **2** (Existence, uniqueness, and explicit expressions for generalized solutions)**.** *Suppose a function $u_{0}$ belongs to the Sobolev space $H^{1}\left[0,h\right]$ . Then:*

(i) *problem* (21) *has a unique generalized solution;*
(ii) *the following formula can be used to calculate the generalized solution:*
(29) $$u\left(y,t\right)=u_{*}\left(y\right)+\sum_{m=1}^{\infty }C_{m}q_{m}\left(y\right)\varphi_{m}\left(t\right),$$
  *where*
(30) $$u_{*}\left(y\right)\overset{\mathrm{def}}{=}-\frac{\xi }{2\mu }y\left(y-h\right)+\frac{\xi h}{2k}\;\;\left(\mathrm{the}\mathrm{steady-state}\mathrm{component}\right),$$
(31) $$q_{m}\left(y\right)\overset{\mathrm{def}}{=}\frac{h}{\eta_{m}}{\left(\frac{h}{2}+\frac{\mu kh^{2}}{\eta_{m}^{2}\mu^{2}+k^{2}h^{2}}\right)}^{-\frac{1}{2}}\sin\left(\frac{\eta_{m}}{h}\;y+\psi_{m}\right),$$
(32) $$\varphi_{m}\left(t\right)\overset{\mathrm{def}}{=}\exp\left(-\frac{\eta_{m}^{2}\mu }{h^{2}+\alpha \eta_{m}^{2}}\;t\right),\;\psi_{m}\overset{\mathrm{def}}{=}\arctan\left(\frac{\mu \eta_{m}}{kh}\right),$$
(33) $$\begin{array}{cc} C_{m} & \overset{\mathrm{def}}{=}\int_{0}^{h}\left(u_{0}^{\prime }\left(y\right)-u_{*}^{\prime }\left(y\right)\right)q_{m}^{\prime }\left(y\right)\;dy\\ \; & \;+\frac{k}{\mu }\left(u_{0}\left(0\right)-u_{*}\left(0\right)\right)q_{m}\left(0\right)+\frac{k}{\mu }\left(u_{0}\left(h\right)-u_{*}\left(h\right)\right)q_{m}\left(h\right), \end{array}$$
  *and the numbers $\left\{\eta_{1},\eta_{2},\ldots \right\}$ are positive roots of the transcendental equation*
(34) $$\cot\eta =\frac{\mu \eta }{2kh}-\frac{kh}{2\mu \eta }$$
  *with respect to η.*

**Proof.**  Following [47], we will construct a generalized solution of (21) using the reduction of the original problem to a Sturm–Liouville problem with Robin-type boundary conditions. The proof of Theorem 2 is derived in five steps.

*Step 1: Passing to a new unknown function*. Consider the function $u_{*}$ , which is defined in (30). Using the representation

$$u\left(y,t\right)=\tilde{u}\left(y,t\right)+u_{*}\left(y\right),$$

we reduce (21) to a new problem:

(35) $$\left\{\begin{array}{cc} \frac{\partial \tilde{u}}{\partial t}-\mu \frac{\partial^{2}\;\tilde{u}}{\partial y^{2}}-\alpha \frac{\partial^{3}\;\tilde{u}}{\partial t\partial y^{2}}=0,\; & \;y\in (0,h),\;t\in (0,T),\\ \mu \frac{\partial \tilde{u}}{\partial y}=k\tilde{u},\; & \;y=0,\;\;t\in (0,T),\\ \mu \frac{\partial \tilde{u}}{\partial y}=-k\tilde{u},\; & \;y=h,\;\;t\in (0,T),\\ \tilde{u}=u_{0}-u_{*},\; & \;y\in (0,h),\;\;t=0 \end{array}\right.$$

with respect to a new unknown function $\tilde{u}$ .

*Step 2: Separation of variables*.

Let us construct nontrivial solutions of problem (35) in the form

$$\tilde{u}\left(y,t\right)=\varphi \left(t\right)\;q\left(y\right).$$

From the first relation of (35), we derive

(36) $$\varphi^{\prime }\left(t\right)\left(q\left(y\right)-\alpha \;q^{\prime \prime }\left(y\right)\right)=\mu \;\varphi \left(t\right)\;q^{\prime \prime }\left(y\right).$$

We are interested in the case when $q-\alpha \;q^{\prime \prime }≢0.$ Indeed, if $q-\alpha \;q^{\prime \prime }\equiv 0$ , then from (36) it follows that $q^{\prime \prime }\equiv 0$ , which is not compatible with the used boundary conditions when q≢0 .

Let $y_{0}$ be a point such that $q\left(y_{0}\right)-\alpha \;q^{\prime \prime }\left(y_{0}\right)\neq 0$ and

$$\lambda \overset{\mathrm{def}}{=}\frac{q^{\prime \prime }\left(y_{0}\right)}{q\left(y_{0}\right)-\alpha \;q^{\prime \prime }\left(y_{0}\right)}.$$

Substituting $y=y_{0}$ into (36), we obtain

$$\varphi^{\prime }\left(t\right)\left(q\left(y_{0}\right)-\alpha \;q^{\prime \prime }\left(y_{0}\right)\right)=\mu \;\varphi \left(t\right)\;q^{\prime \prime }\left(y_{0}\right),$$

whence

(37) $$\varphi^{\prime }\left(t\right)=\lambda \;\mu \;\varphi \left(t\right).$$

From (36) and (37) it follows that

$$\lambda \left(q\left(y\right)-\alpha \;q^{\prime \prime }\left(y\right)\right)=q^{\prime \prime }\left(y\right),$$

which is equivalent to

(38) $$-q^{\prime \prime }\left(y\right)+\frac{\lambda }{1+\lambda \;\alpha }\;q\left(y\right)=0.$$

Moreover, in order to satisfy the boundary conditions from system (35), the following relations must be satisfied:

(39) $$\begin{array}{cc} -\mu q^{\prime }\left(0\right)+kq\left(0\right) & =0, \end{array}$$

(40) $$\begin{array}{cc} \mu q^{\prime }\left(h\right)+kq\left(h\right) & =0. \end{array}$$

System (38)–(40) is a particular case of the well-known Sturm–Liouville problem. The eigenvalues related to problems (38)–(40) are the numbers ${\left(\eta_{m}/h\right)}^{2}$ , with m∈N , where $\eta_{m}$ is a positive root of Equation (34), while the eigenfunctions are defined in (31). Note that these eigenfunctions are chosen such that their norms in the space $H_{k/\mu }^{1}\left[0,h\right]$ are equal to 1 for any m∈N .

*Step 3: Finding the decay-in-time components of a solution*.

The values of the parameter λ are determined from the relation

$$-\frac{\lambda_{m}}{1+\lambda_{m}\;\alpha }={\left(\frac{\eta_{m}}{h}\right)}^{2},\;\;m\in N,$$

whence

$$\lambda_{m}=-\frac{\eta_{m}^{2}}{h^{2}+\alpha \eta_{m}^{2}},\;\;m\in N.$$

Substituting these values into (37), we arrive at the formula

$$\varphi_{m,C}\left(t\right)=C\exp\left(-\frac{\eta_{m}^{2}\mu }{h^{2}+\alpha \eta_{m}^{2}}\;t\right),\;\;C=\mathrm{const},\;\;m\in N,$$

for the decay-in-time components of a solution to the problem under consideration.

*Step 4: Constructing a generalized solution in the series form*. Setting $\varphi_{m}\overset{\mathrm{def}}{=}\varphi_{m,1}$ , we introduce a function ${\tilde{u}}_{m}:\left[0,h\right]\times \left[0,T\right]\to R$ ,

(41) $${\tilde{u}}_{m}\left(y,t\right)\overset{\mathrm{def}}{=}q_{m}\left(y\right)\varphi_{m}\left(t\right),\;m\in N,$$

which satisfies the first three relations of system (35).

Using the function sequence ${\left\{{\tilde{u}}_{m}\right\}}_{m=1}^{\infty }$ , one can construct a generalized solution of problem (35). For this purpose, we consider a function series

(42) $$\sum_{m=1}^{\infty }\ell_{m}\;{\tilde{u}}_{m}\left(y,t\right)$$

with

(43) $$\ell_{m}\overset{\mathrm{def}}{=}{\left(u_{0}-u_{*},q_{m}\right)}_{H_{k/\mu }^{1}\left[0,h\right]},\;m\in N.$$

Let us show that series (42) is uniformly convergent on the set [0,h]×[0,T] . Note that the following statements hold.

- From Proposition 1 it follows that the sequence ${\left\{q_{m}\right\}}_{m=1}^{\infty }$ is an orthonormal basis of the space $H_{k/\mu }^{1}\left[0,h\right]$ .
- The following series
$$\sum_{i=1}^{\infty }\ell_{m}q_{m}\left(y\right)$$
  is convergent in the Sobolev space $H^{1}\left[0,h\right]$ , and hence this series is uniformly convergent on [0,h] .
- For any t∈[0,T] , the sequence ${\left\{\varphi_{m}\left(t\right)\right\}}_{m=1}^{\infty }$ is monotone.
- For any t∈[0,T] and m∈N , we have $0<\varphi_{m}\left(t\right)\leq 1$ .

Applying the generalized Abel criterion (see Proposition 3), we establish the uniform convergence of series (42) on [0,h]×[0,T] .

Further, we define the function $\tilde{u}:\left[0,h\right]\times \left[0,T\right]\to R$ by the formula

$$\tilde{u}\left(y,t\right)\overset{\mathrm{def}}{=}\sum_{m=1}^{\infty }\ell_{m}\;{\tilde{u}}_{m}\left(y,t\right)$$

and show that this function is a generalized solution of problem (35).

Let

(44) $${\tilde{u}}^{\left(m\right)}\left(y,t\right)\overset{\mathrm{def}}{=}\sum_{i=1}^{m}\ell_{i}\;{\tilde{u}}_{i}\left(y,t\right).$$

Clearly, we have the following:

(a) for any m∈N , the function ${\tilde{u}}^{\left(m\right)}$ satisfes the first three relations of system (35);
(b) the sequence ${\left\{{\tilde{u}}^{\left(m\right)}\right\}}_{m=1}^{\infty }$ converges to the function $\tilde{u}$ uniformly on [0,h]×[0,T] as m→∞ .

Moreover, by equalities (41), (43) and (44), one can obtain

(45) $$\begin{array}{cc} {\tilde{u}}^{\left(m\right)}\left(y,0\right) & =\sum_{i=1}^{m}\ell_{i}\;{\tilde{u}}_{i}\left(y,0\right)=\sum_{i=1}^{m}\ell_{i}q_{i}\left(y\right)\\ \; & =\sum_{i=1}^{m}{\left(u_{0}-u_{*},q_{i}\right)}_{H_{k/\mu }^{1}\left[0,h\right]}q_{i}\left(y\right). \end{array}$$

Since the sequence ${\left\{q_{i}\right\}}_{i=1}^{\infty }$ is an orthonormal basis of the space $H_{k/\mu }^{1}\left[0,h\right]$ , from (45) it follows that the sequence ${\left\{{\tilde{u}}^{\left(m\right)}\left(\cdot ,0\right)\right\}}_{m=1}^{\infty }$ converges to the function $u_{0}-u_{*}$ in the space $H_{k/\mu }^{1}\left[0,h\right]$ as m→∞ . Hence,

(46) $${\tilde{u}}^{\left(m\right)}\left(\cdot ,0\right)\to u_{0}-u_{*}\mathrm{in}\mathrm{the}\mathrm{space}H^{1}\left[0,h\right]\mathrm{as}m\to \infty .$$

Combining (46) with statements (a) and (b), we deduce that the function $\tilde{u}$ is a generalized solution of problem (35). Therefore, it can easily be checked that the function *u* defined in (29) is a generalized solution of problem (21).

*Step 5: Uniqueness.* The proof of the uniqueness of the obtained generalized solution can be performed by standard techniques (see, for example, [48]), and hence we omit details.

Thus, the proof of Theorem 2 is complete. □

**Figure 2:**
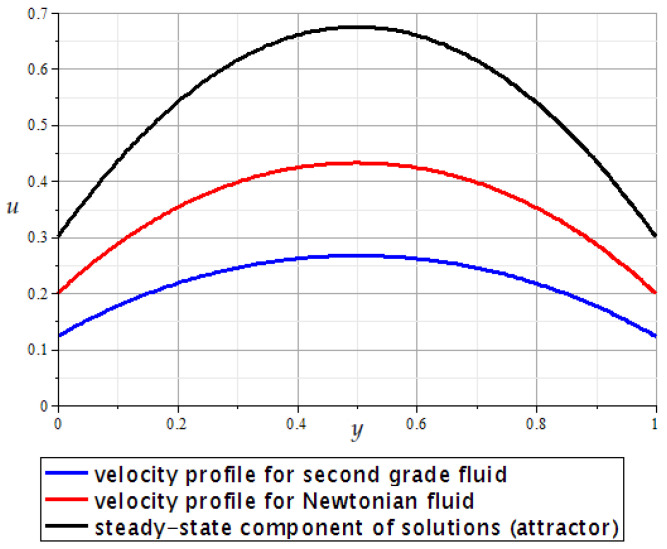
Velocity profiles for the plane Poiseuille flow with h=1 , μ=1 , ξ=3 , k=5 , $u_{0}\equiv 0$ , α=0.2 (second grade fluid), α=0 (Newtonian fluid) at time t=0.2 .

**Remark** **2.** *Using Formulas* (29)–(33)*, it is easy to calculate and visualize the x-component of the velocity for the unsteady Poiseuille flow of both second grade fluids and Newtonian fluids; see, for example, Figure 2.*

## 6. Conclusions

In this paper, we investigated the initial-boundary value problem describing the unsteady Poiseuille flow of a second grade fluid in the channel 0≤y≤h with impermeable solid walls under slip boundary conditions. Applying the concept of generalized solutions, we proved the unique solvability of this problem for any initial data from the Sobolev space $H^{1}\left[0,h\right]$ . Our proof uses the method of separation of variables with a special orthonormal basis consisting of eigenfunctions of the related Sturm–Liouville problem as well as the generalized Abel criterion for uniform convergence of function series. As a result, the problem was solved in a closed form. Namely, the explicit formulas for the flow velocity and the pressure in the channel were established. The obtained analytical solutions contribute to a better understanding of the qualitative features of unsteady flows of polymer fluids and can be used for testing relevant numerical, asymptotic, and approximate analytical methods. Finally, note that the proposed approach is quite universal and can be adapted to solving many other problems related to the channel flows of non-Newtonian fluids with various types of boundary conditions.

## Data Availability

Data are contained within the article.
